# Anticipatory grief of spousal and adult children caregivers of people with dementia

**DOI:** 10.1186/s12904-018-0376-3

**Published:** 2018-11-20

**Authors:** Daphne Sze Ki Cheung, Ken Hok Man Ho, Tsz Fung Cheung, Simon Ching Lam, Mimi Mun Yee Tse

**Affiliations:** 1 School of Nursing, The Hong Kong Polytechnic University, Room GH526, Hung Hom, Kowloon, Hong Kong; 2 School of Nursing, Tung Wah College, Room KPC 16/F, 31 Wylie Road, Homantin, Kowloon, Hong Kong; 3 School of Nursing, The Hong Kong Polytechnic University, Room A133, Hung Hom, Kowloon, Hong Kong; 4 School of Nursing, The Hong Kong Polytechnic University, Room GH523, Hung Hom, Kowloon, Hong Kong; 5 School of Nursing, The Hong Kong Polytechnic University, Room FG425, Hung Hom, Kowloon, Hong Kong

**Keywords:** Anticipatory grief, Dementia, Caregivers, Well-being, Burden

## Abstract

**Background:**

Anticipatory grief (AG) among caregivers of people with dementia is common and has been found to be related to negative health outcomes. Previous studies showed different patterns of AG between spousal and adult children caregivers of people with dementia (PWD) at different stages; however, the levels of such grief are not yet compared. The findings in Western studies are very limited, and inconsistencies have also been found in Asian studies.

**Methods:**

One hundred and eight primary caregivers (54 spousal and 54 adult children) of community-dwelling PWD were recruited from elderly community services sectors in Hong Kong, China through quota sampling. The demographics, AG (measured by the Marwit-Meuser Caregiver Grief Inventory-short form), subjective caregiver burden, and well-being of the participants were assessed. A Functional Assessment Staging Test was used to grade the stages of dementia of the PWD. In this study, those in stages 4 and 5 were regarded as being at an earlier stage, and those in stages 6 and 7 at a later stage of dementia.

The Mann-Whitney U-test and the Chi-square test were used to compare the variables between spousal and adult children caregivers, and the Kruskal–Wallis test was used to compare the outcomes among the sub-groups (spousal caregivers caring for relatives with earlier/later stage dementia; and adult children caregivers caring for relatives with earlier/later stage dementia). A post-hoc analysis was also conducted to identify differences between the sub-groups. Pearson’s correlation was performed to investigate the bivariate relationships among AG, subjective caregiver burden, and well-being.

**Results:**

The results showed that spousal caregivers caring for relatives in a later stage of dementia experienced the highest level of AG and subjective caregiving burden, as compared with spousal caregivers caring for relatives in an earlier stage of dementia and adult children caregivers. Well-being was significantly negatively correlated with AG and subjective caregiver burden, while AG was also significantly correlated with subjective caregiver burden.

**Conclusion:**

This study found that spousal caregivers of relatives in a later stage of dementia have significantly higher levels of AG, warranting special attention and extra support from palliative professionals.

## Background

The anticipatory grief (AG) experienced by dementia caregivers refers to a specific feeling of pre-death grief in response to compound serial losses in the dementia process [1]. Throughout the dementia trajectory, family caregivers witness the gradual cognitive, social, and physical deterioration of significant family members. They report gradual losses as the dementia progresses, including the loss of intimacy and companionship [2–7], or other losses related to the caregiver role such as personal freedom, social or occupational opportunities, recreational opportunities, role identity, and health [8]. A systematic review found that 47 to 71% of dementia caregivers experienced AG [3], and revealed that AG is more common and more serious in dementia caregivers than in caregivers of people with other terminal illnesses [9, 10]. Unresolved AG has a negative impact on the mental health [3, 11], physical health [12], and social relationships of caregivers, who are also at a higher risk of developing complicated and prolonged grief after the death of the care recipients [13, 14]. AG was also found to be associated with a higher likelihood that the care recipient with dementia will be placed in a long-term care setting [15, 16].

The AG experienced in the context of dementia caregiving is more complex than that experienced in providing care to people with other terminal conditions. The gradual and long-lasting physical and cognitive decline of people with dementia (PWD) further complicates the dyadic relationship [17]. According to the Social Exchange Theory [18], the gradual loss of cognitive function in PWD eventually decreases the level of emotional reward experienced by family caregivers, due, for example, to the lack of opportunity to resolve conflicts and share feelings in the dyadic interactions. Therefore, the caregiver expects that there will be a loss of intimacy and mutuality between family members, and that the identity of family members will eventually fade from the consciousness of the care recipient [19]. It is argued that the progressive development of behavioural and psychological symptoms of dementia at different stages and according to different types of dementia will be damaging to the caregiving process as well [20]. The Grief-Stress Model of Caregiving suggests that the losses in a relationship and the losses caused by taking on the caregiving role are what lead to AG [8]. As such, the nature of relationships and the stages of dementia should be taken into consideration in any attempts to address AG.

Among the two major groups of family caregivers, the levels of AG experienced by spouses and adult children at different stages of dementia are still disputed. Three studies conducted in the US and one study conducted in Poland showed that the overall levels of AG were similar between spousal and adult children caregivers [2, 4, 21, 22]. However, these studies did not provide insights on the groups at risk of developing AG at different stages of dementia. In contrast, a Singaporean study showed that both the spousal caregivers and those caring relatives with advanced dementia were more at risk of developing AG, but only 6.9% of the sample in that study consisted of spousal caregivers [23]. Another study, conducted in Hong Kong, also found that spousal caregivers had significantly higher overall AG [24]. However, similar to the Singaporean study, a relatively low portion of spousal caregivers (25%) had been recruited. More importantly, the Hong Kong study might be biased in a way that more than half of the participants received tertiary education and were in middle-class, which may mask the effects of low education and poor socio-economic status as validated risk factors to AG [25]. On the other hand, Zarit argued that caring for a PWD is more burdensome for the adult children than for the spouse of a PWD because of the competing demands on them from a spouse/partner, children, or employment [19]. This ‘sandwich generation’ (i.e., those with older parents and a younger generation to care for) caregivers of PWD has been found to suffer from stress, anxiety, and sadness [26]. According to the Caregiving Stress Model, these competing demands, as modifying factors, may further exacerbate their AG when the dementia of the care recipient becomes more severe.

Trustworthy studies about the AG of populations embedded in the Chinese culture are lacking. It is of paramount importance to compare levels of AG between spousal and adult children caregivers of persons at different stages of dementia in a Chinese population to better determine how to prioritize the allocation of resources to help those in the greatest need. In addition, given that there is a strong Confucian emphasis among Chinese populations on the filial responsibility of children to care for their ill parents, this could affect a person’s attitude towards caregiving, such as their way of appraising their personal sacrifice in dementia caregiving [27]. Lai expressed the view that filial piety is an emotion-focused resource for caregivers coping with the changes or losses resulting from dementia [28]. By comparing AG levels between the spousal and adult children caregivers of people at an earlier or later stage of dementia, this study further explores the association among AG, subjective caregiving burden, and well-being. This will allow for better support to be given to spouses and adult children who have taken on caregiving roles and functions. This study was conducted in Hong Kong, but the findings of this study could potentially be applicable to caregivers in Mainland China, as well as in other Asian countries where filial piety is also emphasized, or in communities of Chinese immigrants all over the world.

## Methods

### Aim

The aims of this study were to compare AG levels between spousal and adult children caregivers of people at earlier or later stages of dementia; and to explore the relationship of the level of AG with subjective caregiving burden and well-being.

### Design

This was a cross-sectional study using quota-sampling.

### Setting and participants

The target population were the spousal or adult children primary caregivers of community-dwelling PWD in Hong Kong. Data were collected from June 2016 to September 2017. The participants had to be either the spouse, cohabiting partner, or adult child of a community-dwelling person who had been diagnosed with any type of dementia; aged 18 or above; and able to understand Chinese. Those who had been diagnosed with a terminal illness or with cognitive impairment were excluded. Four centres providing community elderly services (such as health education, carer support services, meal services, referral services for community resources, etc.) were invited by personal network to refer caregivers to this study. These centres were serving around 200–300 older adults in the neighbourhood, and they were scattered geographically in the Hong Kong territory (two in Kowloon, one in the New Territories, and one in Hong Kong Island).

A quota sampling method was employed to recruit spousal and adult children caregivers at a ratio of 1:1. Each centre was asked to generate a list of elderly members with confirmed diagnosis of dementia, in reverse chronological order of membership registration date. The centre-in-charge then screened and confirmed the eligibility of their primary caregivers with a trained research assistant following the selection criteria. Two lists of caregivers – spousal caregiver and adult children caregivers were generated for each centre. Each centre was asked to invite the first spousal caregiver, then the first adult children caregiver on the lists; and so on until either list was exhausted. If the first adult children caregiver refused to participate, the centre-in-charge would contact the second adult children caregiver on the list, and so on. Once the first invited centre had completed the recruitment, the second, third and fourth centre would invited the caregivers following the same principles until the sample size was reached. An informed written consent to participate in the study was obtained from the participants upon invitation.

The sample size estimation was based on a prior power analysis using G*Power 3.1. There was no prior study examining the difference in anticipatory grief between spousal and adult children caregivers caring people with earlier or later stage of dementia. Based on the latest validation study with samples of spousal and non-spousal caregivers [24], with the power of 0.8, α = 0.05, we estimated that a total of 74 subjects were needed.

### Measures

Data were collected by self-administered questionnaires. A trained research assistant was able to offer help to those participants who required assistance, such as those who were illiterate. Demographic data were collected, including data on gender, age, marital status, education level, income level, religion, relationship with the PWD, and duration of caring for the PWD. Information about the PWD was also collected, including their age, gender, length of time since receiving the diagnosis of dementia, and the stage of dementia in terms of the Functional Assessment Staging Test (FAST) [29]. In FAST, stages 4 to 7 are regarded as the stages of dementia, and with those in stages 4 and 5 are defined as having ‘moderate and moderately severe cognitive decline’ that refers to “mild and moderate dementia” respectively. Those in stages 6 and 7 are regarded as having ‘severe and very severe cognitive decline’ that means “moderately severe and severe dementia” [29]. In this study, we regarded stages 4 or 5 as an earlier stage of dementia, and stages 6 or 7 as a later stage of dementia. The information collected was checked with the centre-in-charge to ensure the accuracy.

### Anticipatory grief

The AG level of the caregivers was measured using a validated Cantonese version of the Marwit-Meuser Caregiver Grief Inventory Short Form (MM-CGI-SF) [24]. This is an 18-item instrument that uses a 5-point Likert scale from 1 (Strongly disagree) to 5 (Strongly agree) to measure grief in three aspects: Personal Sacrifice Burden, Heartfelt Sadness & Longing, and Worry & Felt Isolation, with each aspect being measured using six questions [30]. The Personal Sacrifice Burden subscale is used to assess the experience of individual losses due to caregiving; the Heartfelt Sadness & Longing subscale to examine the intrapersonal reactions to lost relationships; and the Worry & Felt Isolation subscale to determine to the feeling of losing one’s connection with others, which is a feature of a reaction to grief. A higher score indicates a higher level of anticipatory grief. The total score was the sum of the three sub-scores. The internal consistency of this instrument was good to excellent (the Cronbach’s alpha ranged from 0.86–0.94 for the total scores and the subscale scores).

### Subjective caregiver burden

The subjective caregiver burden was measured using the Chinese version of the Zarit Burden Interview (ZBI) [31]. This consists of 22 questions on different aspects of the burden on caregivers. Each question is graded using a 5-point Likert scale, from 0 (never) to 4 (nearly always). A higher score indicates a greater perceived burden. The internal consistency of the instrument was good (split-half correlation coefficient = 0.81) and the inter-rater reliability was excellent (ICC = 0.99).

### Well-being

Caregiver well-being was measured using the Cantonese version of the Personal Well-being Index (PWI) [32]. This is an 8-item instrument that measures subjective well-being using an 11-point Likert scale from 0 (Completely dissatisfied) to 10 (Completely satisfied). The average score of these 8 items was used as an indicator of well-being in this study, with a higher score indicating greater well-being. The internal consistency of the instrument was good (Cronbach’s alpha = 0.80) [33].

### Statistical analyses

IBM SPSS version 23 software was used to manage and analyse the data. Demographic and outcome measurements were presented in terms of means with standard deviations and median with interquartile range (IQR) for continuous data, or in frequencies with percentages for categorical data. Normality of the continuous data was checked with the Kolmogorov-Smirnov test. The differences in demographic variables of caregivers and care-recipients between spousal and adult children caregivers were compared using an independent t-test for normally distributed or the Mann-Whitney U test for non-normality distributed continuous variables, and the Chi-square test for categorical variables. The differences in outcomes variables between spousal and adult children caregivers and among sub-groups (i.e., spousal caregivers caring for relatives with earlier/later stage dementia; and adult children caregivers caring for relatives with earlier/later stage dementia) were compared using an Independent-t test and a One-way ANOVA respectively if the data were normally distributed, otherwise the Mann-Whitney U-test and the Kruskal-Wallis test were used. Post-hoc analysis was performed if there was significant difference in outcome variables among four sub-groups. Univariate associations between AG, well-being, and subjective caregiving burden were examined using Pearson’s correlation or Spearman’s correlation subjected to the normality test result. The level of significance was set at *p* ≤ .05.

## Results

We recruited 108 participants for this study. Table 1 shows the demographics and outcome variables of the participants and the characteristics of the PWD being cared for by the participants. The median age of the spousal caregivers was 72.0 years [IQR = 66.0–81.3], while that of the adult children was 53.5 [IQR = 50.0–57.25] years. Similar to other studies, the majority of the caregivers were female (78.7%). More spousal caregivers than adult children caregivers were living with the PWD. The majority of the spousal caregivers were taking care of a PWD who was male, while most of the adult children caregivers were taking care of a PWD who was female.

**Table 1:** Characteristics of the participants

	All participants (*N* = 108)		Spousal caregiver (*N* = 54)		Adult children caregiver (*N* = 54)		*p*-value
	Mean ± S.D.	Median [25th percentile, 75th percentile]	Mean ± S.D.	Median [25th percentile, 75th percentile]	Mean ± S.D.	Median [25th percentile, 75th percentile]	
Age	62.9 ± 12.6	62.0 [52.0, 72.0]	71.6 ± 10.6	72.0 [66.0, 81.3]	54.2 ± 7.3	53.5 [50.0, 57.25]	<.001
Age of PWD	81.6 ± 7.8	82.0 [77.0, 87.0]	78.3 ± 8.0	78.0 [72.0, 84.25)	84.9 ± 6.0	86.0 [82.0, 90.0]	<.001
No. of years since the diagnosis of dementia	5.4 ± 4.6	4.5 [2.0, 7.0]	5.0 ± 3.3	4.5 [2.0, 8.0]	5.7 ± 5.7	4.5 [2.8, 7.0]	.865
No. of years of being cared for by the participating caregivers	5.2 ± 4.0	4.0 [2.0, 7.0]	5.0 ± 3.5	4.0 [2.0, 7.0]	5.5 ± 4.5	5.0 [2.4, 6.3]	.672
MM-CGI-SF total score	57.7 ± 14.8	57.0 [47.0, 69.8]	64.0 ± 14.7	67.0 [54.5, 76.3]	51.4 ± 12.0	50.0 [45.8, 60.0]	<.001
ZBI total score	43.6 ± 18.2	45.0 [30.0, 56.0]	48.5 ± 18.6	47.5 [33.0, 66.0]	38.8 ± 16.5	40.0 [27.0, 52.0]	.007
PWI total score	6.1 ± 1.7	6.4 [4.9, 7.3]	5.9 ± 1.8	6.3 [4.4, 7.2]	6.4 ± 1.6	6.5 [5.5, 7.4]	.254
Frequency (%)							*p*-value
Gender							.814
Male	23 (21.3%)		12 (22.2%)		11 (20.4%)		
Female	85 (78.7%)		42 (77.8%)		43 (79.6%)		
Marital status							<.001
Never married	21 (19.4%)		2 (3.7%)		19 (35.2%)		
Married	84 (77.8%)		52 (96.3%)		32 (59.3%)		
Separated	1 (0.9%)		0 (0.0%)		1 (1.9%)		
Widowed	2 (1.9%)		0 (0.0%)		2 (3.7%)		
Educational level							<.001
No education	10 (9.3%)		10 (18.5%)		0 (0.0%)		
Primary	28 (25.9%)		26 (48.1%)		2 (3.7%)		
Secondary	43 (39.8%)		14 (25.9%)		29 (53.7%)		
Tertiary	27 (25.0%)		4 (7.4%)		23 (42.6%)		
Religion							.101
No religion	52 (48.1%)		25 (46.3%)		27 (50.0%)		
Worship ancestors	9 (8.3%)		7 (13.0%)		2 (3.7%)		
Taoism/Buddhism	14 (13.0%)		10 (18.5%)		4 (7.4%)		
Christianity/Catholicism	33 (30.6%)		12 (22.2%)		21 (38.9%)		
Monthly income^a^							<.001
< HK$10000 (US$1282)	65 (60.2%)		46 (85.2%)		19 (35.2%)		
HK$10000–19,999 (US$1282–2564)	20 (18.5%)		4 (7.4%)		16 (29.6%)		
HK$20000–29,999 (US$2564–3846)	8 (7.4%)		0 (0.0%)		8 (14.8%)		
HK$30000–39,999 (US$3846–5128)	5 (4.6%)		1 (1.9%)		4 (7.4%)		
HK$40000–49,999 (US$5128–6410)	4 (3.7%)		0 (0.0%)		4 (7.4%)		
≥HK$50000 (US$6410)	6 (5.6%)		3 (5.6%)		3 (5.6%)		
Gender of care-receipt							<.001
Male	48 (44.4%)		41 (75.9%)		7 (13.0%)		
Female	60 (55.6%)		13 (24.1%)		47 (87.0%)		
FAST score							.996
4 or 5 (earlier stage of dementia)	73 (67.6%)		36 (66.7%)		37 (68.5%)		
6 or 7 (later stage of dementia)	35 (32.4%)		18 (33.3%)		17 (31.5%)		
Living with the care-recipient							<.001
Yes	85 (78.7%)		52 (96.3%)		33 (61.1%)		
No	23 (21.3%)		2 (3.7%)		21 (38.9%)		

Remarks: Because of the rounding of decimals, the sum of the percentages might not be 100%. MM-CGI-SF: Marwit-Meuser Caregiver Grief Inventory Short Form; ZBI: Zarit Burden Interview; PWI: Personal Well-being Index; FAST: Functional Assessment and Staging Test

^a^US$1 = HK$7.8; the median monthly household income in 2016 was HK$24,900 (US$3192) [43]

In the Kruskal-Wallis test, significant differences were found among the four sub-groups of caregivers in the overall AG (MM-CGI-SF total score; H = 27.66, *p* < .001) and sub-scores: (a) Personal Sacrifice Burden (H = 16.92, *p* = .001); (b) Heartfelt Sadness and Longing (H = 29.69, *p* < .001); (c) Worry and Felt Isolation (H = 24.32, *p* < .001), and caregiver subjective burden (ZBI total scores; H = 11.94, *p* = .008), but no significant differences were found in well-being (PWI score; H = 6.55, *p* = .880). Post-hoc analysis was followed to investigate which group of caregivers was significantly different from which other groups.

In the post-hoc analysis, spousal caregivers who were caring for people with later stage dementia had a higher MM-CGI-SF total score and Heartfelt Sadness and Longing sub-score as compared with the other three groups (i.e., adult children caregivers who were caring for relatives with earlier/later stage dementia, and spousal caregivers who were caring for relatives with earlier stage dementia). The two MM-CGI-SF sub-scores of Personal Sacrifice Burden, Worry and Felt Isolation, as well as the subjective caregiver burden of spousal caregivers taking care of relatives with later stage dementia were significantly higher than those of the two groups of adult children caregivers, but not significantly different from those of the spousal caregivers who were caring for relatives with earlier stage dementia. Tables 2 and 3 show the results of the comparisons among the groups.

**Table 2:** Comparison of AG, well-being, and burden among different groups

Score	Overall (*n* = 108)	Sp_Early (*n* = 36)	Sp_Late (*n* = 18)	Ac_Early (*n* = 37)	Ac_Late (*n* = 17)	Krusal-Wallis Test statistics	*p*-value
	Median [25th percentile, 75th percentile]						
MM-CGI-SF total score	57.00 [47.00, 69.75]	63.50 [47.00, 72.00]	76.50 [65.00, 79.25]	52.00 [46.00, 60.00]	49.00 [39.50, 57.50]	27.662	**< 0.001**
MM-CGI-SF Personal Sacrifice Burden sub-score	21.00 [16.00, 25.00]	23.00 [16.25, 25.00]	26.50 [21.50, 30.00]	19.00 [16.00, 24.00]	17.00 [15.00, 20.00]	16.917	**0.001**
MM-CGI-SF Heartfelt Sadness & Longing sub-score	19.00 [15.25, 23.00]	20.00 [16.00, 23.00]	26.00 [19.75, 27.50]	17.00 [14.50, 20.00]	18.00 [14.50, 19.50]	29.689	**< 0.001**
MM-CGI-SF Worry & Felt Isolation sub-score	17.25 [13.25, 21.00]	19.50 [15.25, 21.75]	23.00 [17.75, 26.25]	15.00 [13.00, 19.00]	14.00 [11.50, 18.50]	24.323	**< 0.001**
ZBI total score	45.00 [30.00, 56.00]	45.50 [33.00, 59.50]	57.50 [37.00, 71.00]	44.00 [26.50, 52.00]	38.00 [27.50, 50.00]	11.937	**0.008**
PWI total score	6.44 [4.91, 7.34]	6.69 [5.00, 7.59]	5.19 [3.06, 7.03]	6.88 [5.81, 7.50]	5.75 [5.19, 6.94]	6.550	0.088

Remarks: *MM-CGI-SF* Marwit-Meuser Caregiver Grief Inventory Short Form, *ZBI* Zarit Burden Interview, *PWI* Personal Well-being Index. *Sp_Early* Spousal caregivers of a relative with earlier stage dementia, *Sp_Late* Spousal caregivers of a relative with later stage dementia, *Ac_Early* Adult children caregivers of a relative with earlier stage dementia, *Ac_Late*: Adult children caregivers of a relative with later stage dementia

The boldface were significant *p*-value at <.05

**Table 3:** Results of the post-hoc analysis

	Sp_Early (*n* = 36)	Sp_Late (*n* = 18)	Ac_Early (*n* = 37)	Ac_Late (*n* = 17)
MM-CGI-SF total score				
Sp_Early	–			
Sp_Late	*p* = .026	–		
Ac_Early	N.S.	*p* < .001	–	
Ac_Late	N.S.	*p* < .001	N.S.	–
MM-CGI-SF Personal Sacrifice Burden sub-score				
Sp_Early	–			
Sp_Late	N.S.	–		
Ac_Early	N.S.	*p* = .005	–	
Ac_Late	N.S.	*p* = .001	N.S.	–
MM-CGI-SF Heartfelt Sadness and Longing sub-score				
Sp_Early	–			
Sp_Late	*p* = .019	–		
Ac_Early	N.S.	*p* < .001	–	
Ac_Late	N.S.	*p* < .001	N.S.	–
MM-CGI-SF Worry and Felt Isolation sub-score				
Sp_Early	–			
Sp_Late	N.S.	–		
Ac_Early	N.S.	*p* < .001	–	
Ac_Late	*p* = .041	*p* < .001	N.S.	–
ZBI total score				
Sp_Early	–			
Sp_Late	N.S.	–		
Ac_Early	N.S.	*p* = .017	–	
Ac_Late	N.S.	*p* = .012	N.S.	–

Remarks: Data were expressed in *p*-values in the post-hoc analysis with Dunn’s test. *MM-CGI-SF* Marwit-Meuser Caregiver Grief Inventory Short Form, *ZBI* Zarit Burden Interview, *Sp_Early* Spousal caregivers of a relative with earlier stage dementia, *Sp_Late* Spousal caregivers of a relative with later stage dementia, *Ac_Early* Adult children caregivers of a relative with earlier stage dementia, *Ac_Late* Adult children caregivers of a relative with later stage dementia, *N.S.* non-significant

Bivariate correlations among AG, well-being, and subjective caregiving burden were examined using Pearson’s correlation analysis, and the results were shown in Table 4. AG was negatively correlated with well-being (*r* = 0.55, *p* < .001) and positively correlated with subjective caregiver burden (*r* = 0.76, *p* < .001).

**Table 4:** Bivariate correlations between different measures

Measures	1.MM-CGI-SF	2. PWI	3. ZBI
1. MM-CGI-SF	–	−0.551**	0.762**
2. PWI		–	−0.596**
3. ZBI			–

Remarks: MM-CGI-SF: Meuser Caregiver Grief Inventory Short Form; PWI: Personal Well-Being Index; ZBI: Zarit Burden Interview; **p-value < 0.001

## Discussion

To the best of our knowledge, this is the first study to compare the AG, well-being, and burden of spousal and adult children caregivers of people with earlier or later stage dementia; and to explore the relationship among these three variables. First, in general, spousal caregivers experienced a higher level of AG and a greater burden. The overall AG and sub-scale Heartfelt Sadness and Longing of spousal caregivers who were caring for people with later stage dementia were significantly worse than those of the other caregivers. They were also experiencing higher Personal Sacrifice Burden, and Worry and Felt Isolation of AG subscales, as well as the burden higher than the other two sub-groups of adult children caregivers. This novel finding is alarming, and indicates that this group of caregivers warrants more attention. An earlier well-designed validation study conducted by Marwit and Meuser had found similar pattern, i.e., spousal caregivers caring relatives with the most severe stage of dementia (Clinical Dementia Rating Scale Stage 3), experienced the highest overall AG (measured by the 50-item MM-CGI) and the subscales as compared to children caregivers revealed by their mean scores [34]. Echoing the focus group findings of another study by Meuser and Marwit, spousal caregivers of a relative with severe dementia stated that they experienced a full loss of their marriage and acknowledged feeling disconnected from both family and friends [4]. The higher AG that results from caring for a person with profound dementia may be in part due to the awareness that there is no hope of an improvement in that person’s condition, and that their loved one is also approaching the end of his/her life. In addition, the complete loss of the ability to communicate with the PWD may also deepen the sense of a loss of mutuality. Although we did not collect information on the amount of time that the caregivers had spent living with the PWD, spousal caregivers have usually lived with the PWD for a longer time than have the adult children caregivers, and they may have had a longer and closer view of the functional deterioration of the PWD. Another validation study in Hong Kong also found that spousal caregivers were experiencing statistically significant higher AG than non-spousal caregivers of dementia, together with an increasing trend in the AG level when the PWD’s cognitive impairment becoming more severe [24].

Inconsistent findings between Western or Chinese studies were noted when comparing the AG between two types of caregivers. This may be attributed to the cultural difference. In Chinese culture, emotional bonding, interdependence and obligations/responsibilities are strongly emphasized in marital relationship [35]. Chinese spousal caregivers demonstrated characteristics of being inseparable, interdependent, and closely connected to their partners with dementia. They often described their need to be with their spouses: “I felt uneasy without him by my side”, “I would rather stay home with him” [36]. A phenomenological study informed us the transition of the spousal caregivers’ lived experience throughout the trajectory of the disease [37]. The spouse of a PWD changes from ‘being with one’s spouse’ to ‘being alone’. According to the Grief-Stress Model of Caregiving, the loss of intimacy/companionship and the change in relationship dynamic may lead to AG if the losses were appraised as significant [8]. Therefore, the Chinese culture may help to explain why Chinese spousal caregivers caring their partners in advanced dementia were feeling higher level of AG than adult children caregivers, which was not always true in the Caucasian population. We suggest that further research be conducted to confirm whether spousal caregivers’ sense of marital closeness and their perception of their relationship with their spouse changes together with the level of their AG along the trajectory of the disease, or that a qualitative approach be used to explore the needs of this group of caregivers.

Contemporary Chinese culture of filial piety emphasizes the responsibility of adult children to care for their ill parents [38]. In the context of intergenerational caregiving, the sense of filial piety can be framed as positive reappraisal and be a form of emotion-focused coping that are especially suitable for uncontrollable event [39, 40] and lead to more positive caregiver outcomes [41]. There are plenty of literature investigating the effects of traditional Chinese culture on caregiver burden [28, 42], yet little is known about its effects on AG. It is interesting to know how the sense of filial piety protects adult children caregiver from anticipatory grief, even though they are facing challenging demands from work, young children and ageing parent. In addition, with the influence of modernization, the sense of filial obligation is generally decayed [38, 43]. Future research on these areas is suggested.

In this study, we found a significant correlation among AG, well-being, and burden that echoes the findings from the theoretical Grief-Stress Model of Caregiving proposed by Noyes et al. [8]. That model suggests that AG and burden are the outcomes of relationship losses and caregiving losses that may negatively affect the well-being of caregivers. However, this study had a cross-sectional design and the causality among these factors could not be confirmed. Yet this study provided valuable information indicating that the importance of AG cannot be underestimated because of its negative impact on well-being. We also suggest that in a future study we may examine the nature of the losses experienced by caregivers that may lead to AG, in order to better construct interventions.

### Clinical implications

The World Health Organization has suggested that by preventing and relieving suffering, palliative care can improve the quality of life of people with life-threatening illnesses and that of their caregivers [44]. However, only about 14% of patients with any kind of disease who need palliative care are currently receiving it, and over 70% of those who do receive palliative care have cancer or cardiovascular diseases [44]. Dementia is a life-limiting disease that has no cure. The European Association for Palliative Care stresses that PWD and their families need dementia-specific palliative care, and that the provision of psychosocial and spiritual support to both the PWD and their families should be a top priority [45]. Hence, professional caregivers have to understand the needs of families in relation to the suffering that the families experience from AG through the various stages of dementia and should be able to provide bereavement support at any time after the diagnosis [45]. Spousal caregivers are generally older and have a lower level of education, which may affect their ability to seek help. In addition, the grief that a caregiver feels before the death of a PWD is usually not openly acknowledged, publicly mourned, or socially supported, which may cause caregivers to refrain from disclosing their concerns [46]. Embedded in a culture influenced by Confucianism and Buddhism, it is even more difficult for Chinese, especially those from the older generation, to express negative feelings and raw emotions [47]. Health and social care professionals often work with family caregivers of PWD, and we suggest that they be more sensitive in recognizing and addressing the AG of spouses. They can help spouses to articulate their grief and their need for help [24].

To date, few interventions are available to address the AG of caregivers of PWD. The Coaching Intervention developed by MacCourt and team, based on the Caregiver Grief model of Marwit and Meuser, has been found to useful for improving the AG of caregivers of PWD by enhancing their sense of empowerment, ability to cope, and resilience [46]. The intervention consists of eight sessions, including introduction to the transition; dimensions of grief; living with grief; honouring grief; maintaining the self; and enhancing resilience. Although the intervention yielded positive results for both spousal and adult children caregivers, the AG level of the spousal caregivers remained higher post-intervention than that of the adult children caregivers. This may imply that a dementia-specific palliative care intervention may not be ‘specific enough’ for spousal caregivers, because the AG experienced by spouses is so intense. Another rigorously designed study examined the effects of a highly individualized telephone-based Cognitive Behavioural Therapy on AG of dementia caregivers. The results showed that even though the AG level of spousal caregivers was higher than adult children caregivers at baseline, the magnitude of the intervention effect was comparatively similar [48]. This study informs us that spousal caregivers could be as responsive as adult children caregivers if the intervention was highly individualized and specialized. We urge those in the field of dementia care to be alert to the differences between two groups of caregivers and to develop individualized and tailored interventions for them.

### Strengths and limitations

In this study, we applied quota sampling to recruit equal numbers of spousal and adult children caregivers to address the flaws of previous studies [23, 24]. The size of the sample in this study is among the largest of any published study on the AG of caregivers of PWD. However, we recruited caregivers by convenience sampling from four organizations, which might have affected the generalizability of this study, although the four organizations were geographically scattered in different districts in Hong Kong. In the community setting, it is relatively difficult to identify people with later stage dementia, which was a constraint in our efforts to recruit their caregivers for this study.

## Conclusion

This study provided evidence showing that the spousal caregivers of people with later stage dementia were experiencing higher levels of AG than adult children caregivers, leading to a more negative sense of well-being and a higher subjective burden than experienced by the latter. We suggest developing individualized and cultural appropriate interventions that are specific to caregivers of PWD to alleviate their AG, particularly in the Chinese population. It is essential to support and empower practitioners for identifying caregivers at risk, and providing timely and suitable interventions to prevent complications, such as complicated post-death grief.

## Abbreviations

AG: Anticipatory grief
PWD: People with dementia
